# A labeled dataset for AI-based cryo-EM map enhancement

**DOI:** 10.1016/j.csbj.2025.06.041

**Published:** 2025-06-30

**Authors:** Nabin Giri, Xiao Chen, Liguo Wang, Jianlin Cheng

**Affiliations:** a Electrical Engineering and Computer Science, University of Missouri, Columbia, 65211, MO, USA; b NextGen Precision Health Institute, University of Missouri, Columbia, 65211, MO, USA; c Computer Science Department, Hamilton College, Clinton, 13323, NY, USA; d Laboratory for BioMolecular Structure, Brookhaven National Laboratory, Upton, 11973, NY, USA

**Keywords:** Cryo-EM, Cryo-EM map enhancement, Protein structure, Dataset

## Abstract

Cryogenic electron microscopy (cryo-EM) has transformed structural biology by enabling near atomic resolution imaging of macromolecular complexes. However, cryo-EM density maps suffer from intrinsic noise arising from structural sources, shot noise, and digital recording, which complicates accurate model building. While various methods for denoising cryo-EM density maps exist, there is a lack of standardized datasets for benchmarking artificial intelligence (AI) approaches. Here, we present an open-source dataset for cryo-EM density map denoising comprising 650 high-resolution (1-4 Å) experimental maps paired with three types of generated label maps: regression maps capturing idealized density distributions, binary classification maps distinguishing structural elements from background, and atom-type classification maps. Each map is standardized to 1 Å voxel size and validated through Fourier Shell Correlation analysis, demonstrating substantial resolution improvements in label maps compared to experimental maps. This resource bridges the gap between structural biology and artificial intelligence communities, allowing researchers to develop and benchmark innovative methods for enhancing cryo-EM density maps.

## Highlights

• A valuable dataset for training and evaluating AI models to enhance cryo-EM density maps.
• Enhanced maps allow more accurate interpretation of molecular structures and visualization of structural features.
• Labels capture all reference atomic components, including complex elements like rRNA.
• Neighbor-atom labeling overcomes limitations in precise atom-level matching.
• This work bridges AI and structural biology, advancing cryo-EM-based structure determination.

## 1 Introduction

Recent advances in cryogenic electron microscopy (cryo-EM) hardware and software, particularly the development of direct electron detectors and improved image processing algorithms, have enabled the determination of macromolecular complex structures at near-atomic resolution (3 Angstrom (Å) or below). These technological breakthroughs have significantly accelerated the field of structural biology [3], [9], allowing researchers to visualize and understand the intricate details of biomolecules with unprecedented clarity. Cryo-EM density maps are three-dimensional (3D) representations of macromolecular structures, reconstructed from thousands of two-dimensional (2D) projection images captured using transmission electron microscopy under cryogenic conditions. In this process, biological samples in aqueous solution are rapidly flash-frozen to preserve their native state and subsequently exposed to low-dose electron beams during imaging to minimize radiation damage. These 2D projections, captured from various orientations, are then computationally aligned and combined to produce high-resolution 3D density maps. However, this process inherently produces raw images with low signal-to-noise ratios (SNR), significantly complicating the accurate interpretation of structural details. Consequently, the reconstructed 3D cryo-EM density maps suffer from noise-related challenges that obstruct precise determination of atomic arrangements. Given that the ultimate goal of cryo-EM single-particle analysis is to obtain accurate 3D atomic models of macromolecular complexes, these noise-related limitations present notable obstacles to the structure-building process [15].

Noise in cryo-EM data manifests at three distinct stages: First, *structural noise* arises from the surrounding ice matrix. This background structure varies from one molecule to the next and is therefore inherently irreproducible. Conceptually, *structural noise* also encompasses any conformational variations within the molecule itself that are not consistently reproduced across samples. Second, *shot noise* results from the quantum nature of electron radiation, introducing statistical variations in electron detection. Third, *digital noise* emerges during the recording and digitization process; whether from photographic granularity and microdensitometer digitization noise in traditional methods, or from readout noise in modern direct electron detectors. While *shot* and *digital* noise appear as random, dust-like distributions without specific patterns (background noise), *structural noise* exhibits defined shapes with stronger density signals that can particularly puzzle interpretation [4].

The combined effect of these noise sources pass on through every stage of the structural determination pipeline, from initial data collection to final 3D reconstruction of cryo-EM density maps. This noise accumulation significantly hampers the global and local resolution and interpretability of the resulting density maps, making accurate atomic model building challenging. In particular, the noise can complicate the visualization of important features such as side-chain densities and ligand binding sites [10], [12], potentially leading to misinterpretation of structural and functional characteristics [7], [8]. Denoising cryo-EM density maps aims to mitigate these experimental noises, significantly enhancing map interpretability by clarifying the three-dimensional arrangement of atoms. This improved clarity is essential for understanding the structural features of the molecule and helps in uncovering the functional role of imaged biomolecules.

## 2 Material and methods

Recently, several AI-based methods have been developed to enhance the quality and interpretability of cryo-EM density maps, including DeepEMhancer [26], EMReady [18], DeepTracer-Denoising [17], and CryoTEN [27]. A key aspect of training these models is the generation of high-quality label maps. Existing approaches include creating simulated cryo-EM density maps using tools like *pdb2mrc* from the EMAN2 package [28], *pdb2vol* from the Situs package [31], or methods based on Rosetta [11]. These simulated maps, derived from known atomic structures, serve as idealized, noise-free ground truth representations of protein density. Alternatively, some models are trained on label maps generated with LocScale [19] by leveraging atomic models to refine experimental cryo-EM maps [26]. These methods generate a noise reduced maps which considerably helps downstream atomic structure modeling tasks.

A deep learning-based cryo-EM density map enhancement method called EMReady [18] shows significant improvements in atomic model building quality when the enhanced maps are used. The method achieved an average residue coverage of 79.7% for built atomic models, substantially higher than the 64.2% obtained from deposited experimental cryo-EM maps. Furthermore, atomic models built from EMReady-processed maps showed better sequence matching at 50.4%, compared to 31.9% for models derived from deposited experimental maps. Similarly, another recent method called CryoTEN [27] achieved an average residue coverage of 70.74% for built atomic models, higher than the 61.87% obtained from deposited experimental cryo-EM maps. Additionally, atomic models built from CryoTEN showed better sequence matching at 37.38%, compared to 34.37% from deposited experimental maps. These improvements highlight how enhancing the quality and interpretability of cryo-EM density maps benefits both manual interpretation and automated structure-building approaches, including Phenix [2], MAINMAST [29], Cryo2Struct [13], DeepTracer [24], DeepMainmast [30], and ModelAngelo [20].

While various methods have been developed for enhancing and denoising cryo-EM density maps, the target label maps they use differ from one another. For instance, in EMReady [18] the target label map is simulated from its associated PDB structure. DeepEMhancer [26] generated target label maps using LocScale [19]. LocScale utilizes atomic models to locally scale cryo-EM density maps, producing improved versions that serve as high-quality label maps for training deep learning models. What distinguishes LocScale-based targets from simulated maps (like those generated via *pdb2vol* from Situs package [31] or EMReady label maps) is that they operate on experimental maps and incorporate local signal modulation, whereas simulation-based methods generate theoretical, noise-free densities directly from atomic coordinates without referencing the experimental data. Other methods, such as LocalDeblur [25] and LocSpiral [21], differ from LocScale in terms of the enhancement strategy. LocalDeblur uses a Wiener filtering approach guided by local resolution estimates, which must be precomputed separately. LocSpiral applies spiral phase transformation and amplitude normalization to locally improve features. However, unlike LocScale, these approaches do not incorporate atomic models and therefore may not produce supervision labels as closely aligned to structural ground truth. One of the key motivations behind using LocScale in DeepEMhancer is its ability to produce high-quality training targets by utilizing atomic model information. Our prepared dataset takes a similar approach in principle, using atomic models to generate clean, high-resolution simulated maps, but differs in that it produces fully voxel-aligned, noise-free label maps directly using the PDB structure and experimental density maps, without requiring extensive processing of the experimental maps. This ensures that during inference, only the experimental cryo-EM map is needed as input, avoiding the need for calculations like local resolution estimation or atomic model fitting, while still benefiting from atom-level supervision during training.

As cryo-EM technology continues its rapid expansion in structural biology, with applications extending to increasingly complex systems such as membrane proteins, large macromolecular assemblies, and heterogeneous samples, there is a need for datasets that bridge disciplinary boundaries and allow AI practitioners to develop innovative methods for enhancing cryo-EM map quality for better interpretability. The dataset presented in this manuscript addresses this need by providing a comprehensive and standardized resource for the development and evaluation of cryo-EM denoising algorithms. By preparing this dataset, we aim to drive methodological innovation at the intersection of AI and structural biology. Our goal is to accelerate progress in cryo-EM density map interpretation and, ultimately, to advance biomedical research through improved macromolecular structure determination from cryo-EM.

Thus, in this work, we introduce an innovative approach for generating high-quality target label maps for AI-based cryo-EM density map enhancement. When paired with experimental cryo-EM maps as input data, these label maps enable supervised training of deep learning models capable of denoising and enhancing experimental density maps. The trained models can subsequently refine noisy experimental data, improving structural clarity and interpretability through artifact and noise reduction. Our methodology differs by its ability to produce fully voxel-aligned, noise-free label maps by leveraging both PDB structural information and experimental density maps, ensuring optimal spatial correspondence for effective model training.

### 2.1 Data acquisition and preprocessing

#### 2.1.1 Dataset curation

We curated a dataset of high-resolution cryo-EM density maps for single-particle proteins from the Electron Microscopy Data Bank [1] (EMDB), selecting those with resolutions between 1 and 4 Å. The corresponding atomic biological assembly structures were retrieved from the Protein Data Bank [6] (PDB) and used to generate label maps. We refer to the deposited maps obtained from EMDB as experimental cryo-EM density maps, which contain noise that must be identified and removed for effective denoising. To ensure data quality, we applied the following filtering criteria: (1) Removal of maps without a corresponding atomic biological assembly structure in the PDB. (2) Removal of maps missing a resolution value determined by the Fourier Shell Correlation (FSC) 0.143 cut-off score. (3) Removal of redundant cryo-EM density maps associated with the same atomic biological assembly structure.

The FSC was computed using the *phenix.mtriage* function from Phenix software suite [2] by providing the experimental cryo-EM density map and its associated PDB structure as input. After filtering, we obtained a final dataset of 650 cryo-EM density maps, which we used to generate labels for training, validation, and testing of deep learning models for cryo-EM density map denoising.

#### 2.1.2 Experimental map standardization

The experimental cryo-EM density maps in EMDB contain significant variations in density values (e.g., [-2.32, 3.91] and [-0.553, 0.762]) and voxel sizes (e.g., ranging from 0.7 Å to 1.6 Å) due to differences in microscope models, electron doses, detectors, and imaging conditions. Since our approach aims to refine cryo-EM density maps at the voxel level through voxel-wise classification or regression models, we standardized all experimental maps to ensure consistency. Specifically, we standardized the voxel size of all experimental cryo-EM density maps to 1 Å using the resampling function in UCSF ChimeraX [23]. This preprocessing step which standardizes the voxel size, allows the deep learning model to learn meaningful patterns across different cryo-EM density maps, ultimately improving its robustness and accuracy in denoising and structure enhancement.

### 2.2 Label map generation workflow

We generated three types of label maps to train AI-based models for cryo-EM density map enhancement: a regression map capturing density values representing an ideal, noise-free cryo-EM map, and two classification label maps that provide complementary information to improve deep learning model learning during training.

#### 2.2.1 Simulated map generation

The first stage of label map generation involves creating clean, noise-free cryo-EM density maps from atomic biological structures deposited in the Protein Data Bank (PDB) using the *pdb2vol* utility from the Situs package [31]. This real-space convolution software generates simulated volumetric maps directly from atomic coordinates, producing idealized density representations without the noise and artifacts inherent in experimental data. All simulated maps were generated with a standardized voxel size of 1 Å to maintain consistency with the preprocessed experimental cryo-EM density maps. Since the simulated cryo-EM density maps generated using *pdb2vol* utilize only PDB structures without reference to the experimental cryo-EM maps, they do not necessarily share the same spatial origin, dimensions, or voxel grid alignment as the experimental counterparts. Therefore, these simulated maps require further processing to have a precise voxel-wise alignment, which is described in the subsequent section.

#### 2.2.2 Label map generation

While simulated maps provide an idealized, artifact-free representation of cryo-EM density, they often lack precise voxel-wise alignment with their corresponding experimental maps. This alignment is important for computing accurate voxel-wise errors during deep learning model optimization. To address this, we processed the simulated maps to ensure they match the size, voxel spacing, and voxel-wise alignment with the experimental maps. To achieve this, we created three empty mask maps (with all voxel values initialized to zero), each matching the dimensions of the experimental cryo-EM density map, to store label information. The mask map created is a 3D grid, where the location of each voxel is determined by indices (i,j,k). But the corresponding PDB structure used to label the voxels are in a 3D coordinate system (x,y,z). Therefore, we calculated the corresponding indices of each atom in the mask map from it's atomic coordinates using the Formula (1), where (i,j,k) are the grid indices of the atom in the mask map, (x,y,z) are the coordinates in the protein structure, $origin_{x},origin_{y},origin_{z}$ are the origin of x,y,z axis respectively found in the experimental cryo-EM density map, and $voxel_{x},voxel_{y},voxel_{z}$ are the voxel size of x,y,z axis respectively found in experimental cryo-EM density map. To ensure consistency, voxels are labeled only when the converted (i,j,k) indices correspond to valid positions in both the simulated map (generated in the previous step using *pdb2vol*) and the experimental cryo-EM density map. This dual-validation approach guarantees that labeled voxels represent structurally relevant regions present in both the simulated and experimental density cryo-EM maps. Following this strategy, we create three label maps:

• **Regression Label Map:** This map contains density values copied from the simulated cryo-EM density map. At each voxel position corresponding to an atom in the PDB structure, we assign the density value from the simulated map, providing a noise-free target for regression-based learning.
• **Classification Label Map:** This binary classification map distinguishes structural elements from background. We assign a value of 1 to voxels corresponding to atomic coordinates present in PDB while background (non-atomic) voxels remain at 0.
• **Atom Type Classification Label Map:** This multi-class map differentiates between atom types, enabling the model to learn chemical specificity. We assign distinct values to voxels based on the corresponding atom type: C*α* (1), C*β* (2), carbonyl carbon (3), oxygen (4), and nitrogen (5) while other voxels remain at 0.

One key idea in our labeling method is how to handle neighboring voxels. When we convert coordinates to grid positions, we change from floating point numbers to integer numbers. This conversion can cause us to lose some accuracy in finding the exact voxel location for each atom. The calculated position might not perfectly match where the atom should be placed in the density map. To fix this problem and make sure we capture the structure properly, we label not just the single voxel found using Formula (1), but also the voxels around it. This approach helps correct for small errors in the conversion process and gives us a better representation of where atoms are located in the 3D grid.

(1) $$i=\lceil \left(\frac{\left\lfloor (z-origin_{z}\right)}{voxel_{z}}\right);j=\lceil \left(\frac{\left\lfloor (y-origin_{y}\right)}{voxel_{y}}\right);k=\lceil \left(\frac{\left\lfloor (x-origin_{x}\right)}{voxel_{x}}\right)$$

As such to improve the structural context, we extended the labeled regions by identifying neighboring voxels within a 6 Å radius of each atomic coordinate. This radius was selected based on analysis of electron density distribution in high-resolution cryo-EM maps and users can change this radius if needed from the source code provided in the GitHub repository. The approach for handling these neighboring voxels varies by label map type:

• For the **Regression Label Map**, neighboring voxels are assigned corresponding values from the simulated cryo-EM density map.
• For the **Classification Label Map**, neighboring voxels are assigned a distinct value of 2, differentiating them from both the central atomic positions (value 1) and the background (value 0). This three-class approach allows the model to learn regions between atoms and background.
• For the **Atom Type Classification Label Map**, we maintain the original atomic type labeling only for voxels corresponding directly to atom positions, as the chemical identity of neighboring voxels cannot be unambiguously determined.

By providing complementary information across different label types, we allow deep learning models to learn diverse aspects of molecular structure, ultimately leading to more robust and accurate way to enhance experimental cryo-EM density maps. These label maps can be used individually or in combination during deep learning model training, allowing for flexible architecture design and task-specific optimization strategies.

### 2.3 Data availability and information

To keep the generated data files permanent, we published all data to the Harvard Dataverse (https://doi.org/10.7910/DVN/CI0J2B), an online data management and sharing platform with a permanent Digital Object Identifier number. Our dataset consists of the following components, curated to support the development and validation of deep learning models for cryo-EM density map enhancement:

#### 2.3.1 Experimental cryo-EM density maps

We compiled 650 high-resolution (1-4 Å) experimental maps from EMDB, standardized to 1 Å voxel size. These maps represent diverse structural classes including soluble proteins, membrane proteins, protein-nucleic acid complexes, and macromolecular assemblies. Each map is stored in MRC format with associated metadata including resolution and PDB accession codes. The maps can be read using mrc package available in Python and the maps can be visualized using UCSF ChimeraX. The naming convention of this map is *pdbID.mrc*.

#### 2.3.2 Atomic biological assembly structures

For each experimental map, we obtained the corresponding biological assembly atomic structure from PDB. These complete assemblies ensure full occupancy of the experimental density maps and serve as the foundation for both label generation and quality validation. The files can be visualized using UCSF ChimeraX. The naming convention of this map is *pdbID.pdb1*.

#### 2.3.3 Simulated cryo-EM density maps

Using the *pdb2vol* utility from Situs, we generated idealized, noise-free density maps from the atomic structures. The maps can be read using mrc package available in Python and the maps can be visualized using UCSF ChimeraX. The naming convention of this map is *pdbID_situs_simulated.mrc*.

#### 2.3.4 Label maps

As described in the Label Map Generation section, we prepared three types of label maps for deep learning training purposes. These label maps follow a naming convention: *pdbID_regression_situs.mrc* for regression maps, *pdbID_classification_types_situs.mrc* for atom type classification maps, and *pdbID_classification_situs.mrc* for general classification maps. The maps can be read using mrc package available in Python and the maps can be visualized using UCSF ChimeraX. All data records maintain consistent dimensions and spatial alignment with their corresponding experimental maps, ensuring proper voxel-to-voxel correspondence for training deep learning models in supervised learning manner. The dataset is organized by PDB identifiers, with supplementary metadata files providing resolution information and quality metrics.

## 3 Technical validation

To objectively validate the quality of our regression label maps compared to the experimental maps, we used Fourier Shell Correlation (FSC). For each map in our dataset (*n = 650*), we computed FSC using the *phenix.mtriage* tool from the Phenix software suite [2]. We analyzed the stringent FSC 0.5 criterion (indicating higher confidence in map-model agreement), using unmasked analyses to evaluate global map quality. The unmasked map-model FSC-0.5 is a measure of the agreement between an experimental density map and a model-calculated density map, assessed at a FSC value of 0.5, without using a mask. Essentially, it indicates how well the atomic model fits the experimental data, and it's calculated without any spatial restrictions on the comparison. In cryo-EM, low resolution value indicates high-resolution map where the fine atomic details, side chains visible clearly.

**Fig. 1:**
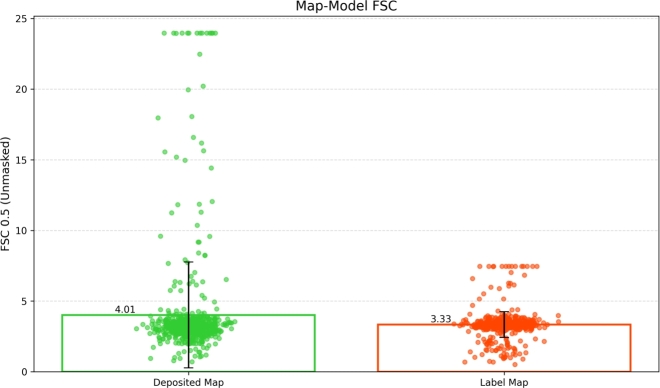
The mean FSC 0.5 unmasked for deposited map and regression label map is 4.01 Å and 3.33 Å, respectively.

Fig. 1 presents a comprehensive scatter plot comparison of resolution metrics between the deposited experimental maps and our regression label maps. The mean unmasked FSC 0.5 resolution improved from 4.01 Å for experimental maps to 3.33 Å for regression label maps, demonstrating a 16.9% enhancement. The distribution pattern shows that regression label maps (BrickRed) maintain more consistent quality at this higher confidence threshold, with experimental maps (green) showing variability. The box plots in highlight the statistical significance of these improvements, with minimal overlap between the distributions. Notably, the upper quartile bound for the regression label maps falls below the mean of the experimental maps in both FSC metrics, emphasizing the consistent better resolution of the label maps.

**Fig. 2:**
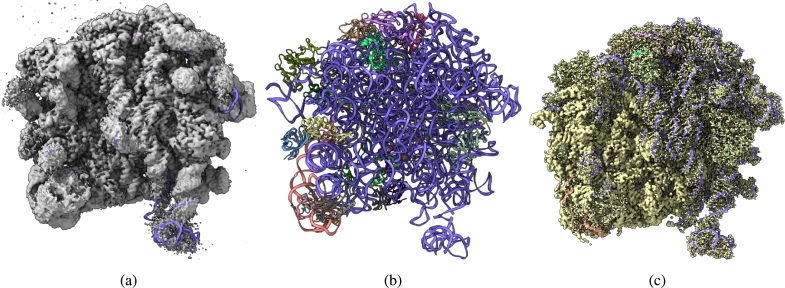
(a) Overlay of the deposited experimental cryo-EM density map (EMD-11900) in grey with dimensions of 308 × 308 × 308 and a voxel size of 1 × 1 × 1 Å, visualized at the recommended contour level of 0.0037 (1.1 *σ*), along with its corresponding biological atomic structure (PDB Code: 7ASM). The Fourier Shell Correlation (FSC) at 0.5 (unmasked) is 2.43 Å. (b) The atomic structure of the protein (PDB Code: 7ASM). (c) The regression label map in yellow with dimensions of 308 × 308 × 308 and a voxel size of 1 × 1 × 1 Å, overlaid with the known biological atomic structure (PDB Code: 7ASM), with an FSC at 0.5 (unmasked) of 1.27 Å.

**Fig. 3:**
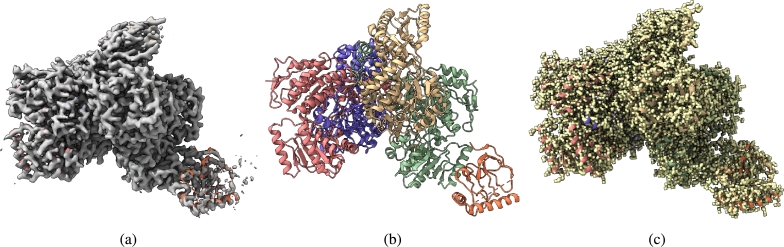
(a) Overlay of the deposited experimental cryo-EM density map (EMD-33113) in grey with dimensions of 283 × 283 × 283 and a voxel size of 1 × 1 × 1 Å, visualized at the recommended contour level of 0.0178 (4.9 *σ*), along with its corresponding biological atomic structure (PDB Code: 7XC6). The Fourier Shell Correlation (FSC) at 0.5 (unmasked) is 4.16 Å. (b) The atomic structure of the protein (PDB Code: 7XC6). (c) The regression label map in yellow with dimensions of 283 × 283 × 283 and a voxel size of 1 × 1 × 1 Å, overlaid with the known biological atomic structure (PDB Code: 7XC6), with an FSC at 0.5 (unmasked) of 3.24 Å.

**Fig. 4:**
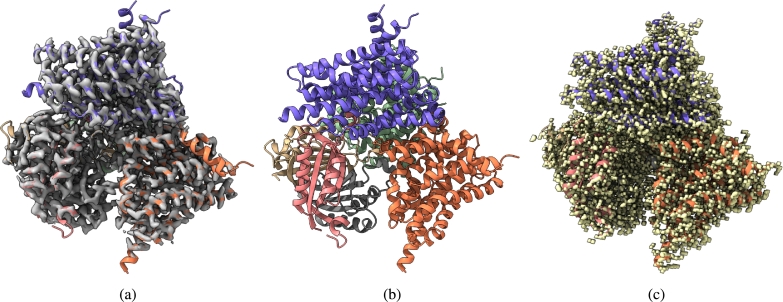
(a) Overlay of the deposited experimental cryo-EM density map (EMD-31135) in grey with dimensions of 258 × 258 × 258 and a voxel size of 1 × 1 × 1 Å, visualized at the recommended contour level of 0.05 (11.5 *σ*), along with its corresponding biological atomic structure (PDB Code: 7EGK). The Fourier Shell Correlation (FSC) at 0.5 (unmasked) is 7.93 Å. (b) The atomic structure of the protein (PDB Code: 7EGK). (c) The regression label map in yellow with dimensions of 258 × 258 × 258 and a voxel size of 1 × 1 × 1 Å, overlaid with the known biological atomic structure (PDB Code: 7EGK) with an FSC at 0.5 (unmasked) of 3.23 Å.

**Fig. 5:**
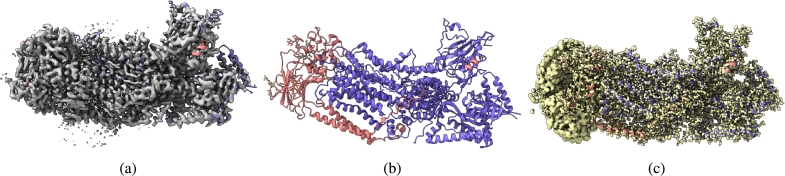
(a) Overlay of the deposited experimental cryo-EM density map (EMD-23075) in grey with dimensions of 231 × 231 × 231 and a voxel size of 1 × 1 × 1 Å, visualized at the recommended contour level of 0.018 (4.3 *σ*), along with its corresponding atomic structure (PDB Code: 7KYC). The Fourier Shell Correlation (FSC) at 0.5 (unmasked) is 2.86 Å. (b) The atomic structure of the protein (PDB Code: 7KYC). (c) The regression label map in yellow with dimensions of 231 × 231 × 231 and a voxel size of 1 × 1 × 1 Å, overlaid with the known biological atomic structure (PDB Code: 7KYC) with an FSC at 0.5 (unmasked) of 1.56 Å.

**Fig. 6:**
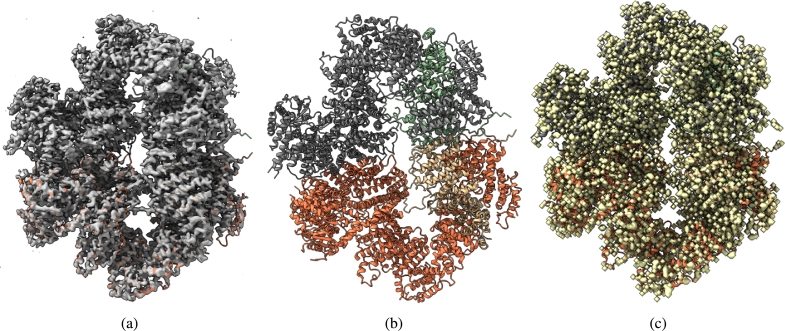
(a) Overlay of the deposited experimental cryo-EM density map (EMD-11055) in grey with dimensions of 347 × 347 × 347 and a voxel size of 1 × 1 × 1 Å, visualized at the recommended contour level of 1.6 (4.9 *σ*), along with its corresponding biological atomic structure (PDB Code: 6Z2W). The Fourier Shell Correlation (FSC) at 0.5 (unmasked) is 6.32 Å. (b) The atomic structure of the protein (PDB Code: 6Z2W). (c) The regression label map in yellow with dimensions of 347 × 347 × 347 and a voxel size of 1 × 1 × 1 Å, overlaid with the known biological atomic structure (PDB Code: 6Z2W) with an FSC at 0.5 (unmasked) of 3.32 Å.

**Fig. 7:**
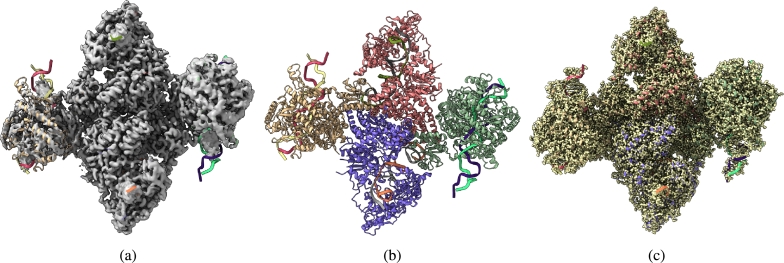
(a) Overlay of the deposited experimental cryo-EM density map (EMD-23461) in grey with dimensions of 526 × 526 × 526 and a voxel size of 1 × 1 × 1 Å, visualized at the recommended contour level of 0.18 (8.2 *σ*), along with its corresponding biological atomic structure (PDB Code: 7LO5). The Fourier Shell Correlation (FSC) at 0.5 (unmasked) is 6.52 Å. (b) The atomic structure of the protein (PDB Code: 7LO5). (c) The regression label map in yellow with dimensions of 526 × 526 × 526 and a voxel size of 1 × 1 × 1 Å, overlaid with the known biological atomic structure (PDB Code: 7LO5) with an FSC at 0.5 (unmasked) of 3.5 Å.

Fig. 2, Fig. 3, Fig. 4, Fig. 5, Fig. 6, Fig. 7 show the experimental cryo-EM density map and the regression labeled cryo-EM density map for visual interpretation. These results provide evidence that our regression label maps provide improved structural representation of the macromolecule compared to the deposited experimental maps. The enhanced resolution and consistency make them ideal targets for training deep learning models aimed at denoising and refining experimental cryo-EM density maps. By learning to transform noisy experimental data toward these higher-quality representations, our models can effectively enhance structural interpretability and in turn more accurate *de novo* atomic modeling [15].

## 4 Discussion

The dataset presented in this work is specifically designed for training and evaluating deep learning models aimed at improving the clarity and interpretability of cryo-EM density maps. By enhancing cryo-EM map quality, these models enable more accurate 3D atomic structure modeling from experimental cryo-EM density maps [15]. Our dataset supports supervised learning approaches through three complementary training paradigms. First, the regression labels paired with experimental cryo-EM density maps enable models to remove experimental artifacts and noise while preserving essential structural features. Second, the classification labels allow deep learning models to distinguish protein structural components from background regions. Finally, the atom-type classification labels support the development of models capable of identifying specific atomic elements within cryo-EM density maps, providing detailed chemical information for structure determination.

We also note that there are other approaches to cryo-EM density map enhancement which have shown promises, particularly self-supervised methods such as Noise2Noise [22] and its derivatives, which have gained attention in both cryo-EM [5] and broader imaging communities. These methods offer the compelling advantage of learning denoising patterns without requiring clean ground-truth data. Within the cryo-EM domain, variants of this self-supervised paradigm utilize independent reconstructions from split datasets (e.g., half-maps) to train models, thereby circumventing the need for idealized or simulated reference labels. Despite these advances in self-supervised learning, supervised methods continue to be widely adopted and often preferred when high-quality reference labels are available. Supervised approaches typically demonstrate stability and output quality, particularly when trained on carefully aligned, voxel-wise datasets. However, the principal limitation of supervised methods lies in their dependency on well-prepared target maps, a requirement that our dataset is specifically designed to address. By providing fully voxel-aligned, noise-free label maps, our approach bridges this gap and enables the development of robust supervised models for cryo-EM density map enhancement.

For optimal results when training deep learning models with this dataset, we recommend that users split the dataset based on resolution, as described in Cryo2StructData [16], rather than using a random split. To improve memory efficiency, an alternative approach is to generate 3D sub-cubes (e.g., 64 × 64 × 64) from the cryo-EM maps instead of processing entire volumes at once as used in Cryo2Struct [13]. We also encourage users to consider a multi-task learning approach, training on multiple label types to improve feature learning and generalization such as shown in [14]. By following these guidelines, researchers can effectively leverage this dataset to develop robust deep learning models for cryo-EM map enhancement, ultimately advancing structural biology research through improved map interpretation and atomic modeling.

## 5 Conclusion

In this study, we presented an approach to generate label mask maps for experimental cryo-EM density maps. These label maps allow to train deep learning models for density map enhancement by identifying and eliminating experimental artifacts that hinder accurate structure modeling.

While we acknowledge that the deposited atomic structures may lack certain specialized components such as ligands, glycans, post-translational modifications, and lipids which might potentially effect the denoising process. Nevertheless, as illustrated in Fig. 2, the rRNA structural components modeled in the atomic structure were successfully identified and labeled, which confirms that our labeling process effectively captures all components present in the reference atomic structures. Furthermore, by labeling the neighbors of atoms, we mitigate issues related to exact matching requirements.

This dataset bridges structural biology and AI, allowing AI practitioners to develop deep learning models that refine and enhance the visibility of structural details in cryo-EM density maps. These improvements will ultimately advance both manual and automated structure modeling from cryo-EM data, increasing our ability to determine macromolecular structures with higher confidence and precision for biomedical research. In the future, we plan to develop and train advanced deep learning methods on this dataset to enhance cryo-EM density maps and apply them to improve atomic model building from cryo-EM data.

## Code availability

The source code and the instructions to reproduce the dataset is provided in the GitHub repository accessible at https://github.com/BioinfoMachineLearning/denoisecryodata.git. To keep the generated data files permanent, we published all data to the Harvard Dataverse (https://doi.org/10.7910/DVN/CI0J2B), an online data management and sharing platform with a permanent Digital Object Identifier number.

## CRediT authorship contribution statement

**Nabin Giri:** Writing – review & editing, Writing – original draft, Visualization, Validation, Methodology, Formal analysis, Data curation. **Xiao Chen:** Writing – review & editing, Writing – original draft, Visualization, Validation, Methodology, Formal analysis, Data curation. **Liguo Wang:** Supervision, Formal analysis. **Jianlin Cheng:** Writing – review & editing, Supervision, Methodology, Formal analysis, Conceptualization.

## Declaration of Competing Interest

The authors declare no competing interests.
